# The Future Dentist

**Published:** 1900-08-15

**Authors:** S. M. Fowler


### THE FUTURE DENTIST.

BY S. M. FOWLER, D.D.S.

---

It had never occurred to me that I was endowed with any special prophetic powers, and when our president asked me to tell the members of the Michigan Dental Association what the future dentist is to be I immediately went into a trance. I tried my best to picture the future dentist sitting at the foot of his stairs, telling his patients, as they filed past in one continuous stream, where to turn to the left—his wife in the office to take the cash, while he watched the parade go by.

But my visions were always incomplete, and as soon as I got my future dentist comfortably located where he had the best view of the parade there always appeared the old familiar quotation, "He that by the plow would thrive, himself must either hold or drive," and so I had to take him away from his comfortable seat and the parade and put him where he belonged—in his office.

The only way we can judge what the future of any organization will be is to inquire into its past history, note what advancement has been made, and then we can with some degree of accuracy predict what may reasonably be expected of it in the future.

It is unnecessary for me to go into the early history of the profession, as you are all as well acquainted with its early struggles as I am. Notwithstanding the antiquity of dentistry, it was not until within the last fifty years that it was recognized as a profession, and until a relatively short time ago the practice was mainly in the hands of those who were without professional education or social standing. Why, since I have been in the profession (twelve years) I have had my barber declare to me in all candor that he thought there was more to my "trade" than there was to his (a declaration that makes a young D.D.S. feel the importance of his calling). But this was early in my professional career, and to-day we find the masses fully alive to the fact that the man who practices dentistry must be in every sense a bright, educated and scientific man. I venture the assertion (and it is fully appreciated by the public) that none of the liberal professions have so few poorly qualified members as our own.. Please do not take this as an admission on my part that the principles of dentistry are so easy that any one can readily master them. The day is past when the young man who considers his education insufficient to permit him to enter the legal, medical or teachers' profession, becomes either a dentist or a preacher. Having never applied for admission to the latter profession, I do not know just what advancement they are making, but to-day we find the young man who has a notion of studying dentistry examining himself to find if he has sufficient education to take up the study.

And right here I want to touch upon a subject which has occurred to me many times, and which I fear will be a delicate subject and one which I expect will call forth a storm of criticism. In looking over the requirements for admission to our dental colleges I find that the applicant must present to the faculty proof that he is a graduate of some recognized academy or high school, or must pass an examination in the following studies: English, arithmetic, algebra, geometry, history, physics, Latin, botany, zoology, physical geography and physiology. Now, any one who has paid any attention to the matter knows that these requirements are not enforced. I know of several who entered college last year who are utterly unable to comply with these requirements. In one case the young man has never passed his eighth grade examinations. In another case the gentleman has not attended school since he was fifteen years of age; he is now thirty-five, and you can imagine his literary attainments when you picture a boy of fifteen leaving school twenty years ago, and yet these same gentlemen matriculated in two of our foremost colleges; and so, I ask you, are dental colleges doing their full share for the advancement of our profession? We have recently heard our State Board of Examiners in Dentistry severely scored for granting permission to practice dentistry in this State to men who were not qualified, and don't you think it time to get after the faculties of dental colleges who are matriculating men who can not possibly pass the required entrance examination?

Do not these methods savor of the commercial way of grasping for the almighty dollar? In the one case, the only motive I can imagine, is to have their next catalogue show the names of more students than some of its rival institutions, and in the other case the motive must be to show to the stockholders of the institution that their investment is paying, and probably if it did not pay the management would be put into more businesslike hands. I have no fault to find with the stockholder of any institution expecting his investment to pay, but I do find fault with the faculty of a dental college—those to whom we must look for the advancement of our profession—admitting students who cannot comply with the requirements.

You may argue that some of these same students make better and more practical dentists than many of those with the highest literary attainments. True, but why have *any* requirements unless they are strictly observed?

But I will stop finding fault, as most of our dental students to-day are at least high school graduates. There are but very few of the other class to which I have referred, and I hope for the early elimination of those few. I have no fear for the future of a profession whose members are required to graduate from our high schools before taking up the study of that profession.

The high schools in many of the larger cities of our State have of late years added manual training departments, making it a part of the high school course and requiring each student to take the manual training course before he can graduate. The influence of such a department can not be overestimated in finding out the adaptability of a young man for the different trades and professions, and is sure to have a direct influence on the dental profession. When manual training is made a part of the course in all our high schools, and all who matriculate in the dental colleges are required to graduate from our high schools, the young men who take up the study of dentistry will have that happy combination so essential to the successful dentist—the necessary literary education combined with a trained, natural, mechanical genius. And so I predict that in the near future the young man who enters the profession of dentistry will do so because he is naturally adapted for the work. He will enter the work knowing that he is going to be successful. The profession in general will be composed of men better prepared for their life work, men who have a better appreciation of the responsibilities they are to assume, and men who will raise the already high standard of our profession. When that time comes, when only those who have minds trained by the hard study and deep thought necessary to complete a literary education are admitted into the dental colleges, then we can look for the greatest advancement in dentistry.

My future dentist is going to be a man who has first qualified himself by a thorough education. He is going to take up the study of dentistry because his work in the manual training department of his high school has shown him that he is particularly adapted for the work. He is going to be honest, for he will fully realize that the interests of the dentist and patient are mutual and that when he is working for the interest of the patient, he is also working for his own best interests.

